# Design of the Bottom-up Innovation project - a participatory, primary preventive, organizational level intervention on work-related stress and well-being for workers in Dutch vocational education

**DOI:** 10.1186/1471-2458-13-760

**Published:** 2013-08-15

**Authors:** Roosmarijn MC Schelvis, Karen M Oude Hengel, Noortje M Wiezer, Birgitte M Blatter, Joost AGM van Genabeek, Ernst T Bohlmeijer, Allard J van der Beek

**Affiliations:** 1 Netherlands Organization for Applied Scientific Research TNO, P.O. Box 718, 2130 AS Hoofddorp, The Netherlands; 2 Body@Work, Research Center on Physical Activity, Work and Health, TNO-VU/VUmc, The Netherlands; 3 Department of Psychology, Health and Technology, University of Twente, Enschede, The Netherlands; 4 Department of Public and Occupational Health, The EMGO+ Institute for Health and Care Research, VU University Medical Center, Amsterdam, The Netherlands

**Keywords:** Stress management, Controlled trial, Need for recovery, Vitality, Occupational self-efficacy, Teachers

## Abstract

**Background:**

In the educational sector job demands have intensified, while job resources remained the same. A prolonged disbalance between demands and resources contributes to lowered vitality and heightened need for recovery, eventually resulting in burnout, sickness absence and retention problems. Until now stress management interventions in education focused mostly on strengthening the individual capacity to cope with stress, instead of altering the sources of stress at work at the organizational level. These interventions have been only partly effective in influencing burnout and well-being. Therefore, the “Bottom-up Innovation” project tests a two-phased participatory, primary preventive organizational level intervention (i.e. a participatory action approach) that targets and engages all workers in the primary process of schools. It is hypothesized that participating in the project results in increased occupational self-efficacy and organizational efficacy. The central research question: is an organization focused stress management intervention based on participatory action effective in reducing the need for recovery and enhancing vitality in school employees in comparison to business as usual?

**Methods/Design:**

The study is designed as a controlled trial with mixed methods and three measurement moments: baseline (quantitative measures), six months and 18 months (quantitative and qualitative measures). At first follow-up short term effects of taking part in the needs assessment (phase 1) will be determined. At second follow-up the long term effects of taking part in the needs assessment will be determined as well as the effects of implemented tailored workplace solutions (phase 2). A process evaluation based on quantitative and qualitative data will shed light on whether, how and why the intervention (does not) work(s).

**Discussion:**

“Bottom-up Innovation” is a combined effort of the educational sector, intervention providers and researchers. Results will provide insight into (1) the relation between participating in the intervention and occupational and organizational self-efficacy, (2) how an improved balance between job demands and job resources might affect need for recovery and vitality, in the short and long term, from an organizational perspective, and (3) success and fail factors for implementation of an organizational intervention.

**Trial registration number:**

Netherlands Trial Register NTR3284

## Background

The Dutch government aspires a top five position in the global rankings for education and science [1], to ensure the competitive power of the Dutch economy. Improving the educational quality is crucial to achieve this ambition. Undisputedly, teachers and their managers play an important role in maintaining and improving the quality of education [2]. However, almost one in five workers in the Dutch educational sector (18%) suffers from work-related stress complaints, compared to one in eight workers in the Dutch working population (13%) [3]. Work-related stress is an important cause for mental health problems, such as burnout. Burnout is associated with reduced work performance (e.g. [4,5]) and its high prevalence in the educational sector thus interferes with the Dutch government’s ambition.

### Work-related stress as a major problem

Burnout, as an ultimate outcome of work-related stress, is considered a prolonged response to chronic emotional and interpersonal stressors in the work context, characterized by emotional exhaustion, depersonalization and reduced personal accomplishment [6]. The work context comprises two specific sets of characteristics that influence burnout and well-being: job demands and job resources. Job demands are generally considered the physical, social or organizational aspects of the job that require sustained physical or psychological effort [7]. Job resources are the physical, social or organizational aspects of the job that may reduce job demands, help to achieve goals and stimulate learning and development [7]. A job demand, such as dealing with students with special needs, will turn into a stressor over time if job resources, such as coworker support, are insufficient or lacking [8,9]. In the educational sector job demands have intensified at rapid pace [10], while job resources remained the same. For example, the student-teacher ratio has increased [11]; students with special needs have been integrated in the regular classes [12]; the number of accountability measures has grown, leading to numerous administrative tasks and consequent paperwork [13]; and several school reforms have been implemented in the educational sector, often even overlapping [14]. It seems likely that this intensification of job demands has contributed to the current burnout rates.

### Consequences of work-related stress

Work-related stress may show as decreased vitality and increased need for recovery. These precursors of burnout have been associated with several other negative organizational outcomes, for example sickness absence and retention problems. First, sickness absence rates are relatively high in the educational sector [3]. More often than in other sectors, workers in education consider their absence a result of emotionally demanding and stressful work [3]. If a teacher falls ill, the work is often temporarily accounted for by his or her colleagues, thereby increasing the workload (i.e. a job demand) for this colleague while job resources remain the same. This practice, although not in line with sickness replacement regulations in Dutch schools, disturbs the equilibrium between job demands and job resources of healthy colleagues. Second, a large number of teachers retire before reaching the official retirement age [15]. Between 45% [16] and 70% [17] of early retirements in teachers is accounted for by psychosomatic illness and psychological problems. Furthermore, approximately half of all novice teachers leave the sector within their first five years, as noted in a North American study [18]. Retention of both novice and experienced teachers is thus a challenge with societal implications. Burnout rates, sickness absence and lower retention rates sum up to a reduced employability of the work force, which is costly. In The Netherlands alone, work days lost due to presenteeism and sickness absence associated with mental health problems summed up to 2.7 billion Euros in 2008 [19,20]. There is thus an urgent need for stress management interventions in the workplace. Ideally these interventions alter precursors of burnout, such as need for recovery and reduced vitality.

### Interventions in education: individual-focused and secondary preventive

Stress management interventions can be classified as primary, secondary or tertiary prevention. Primary preventive interventions aim to alter the sources of stress at work (e.g. [21]). Secondary preventive interventions aim to reduce stress symptoms before they lead to health problems (e.g. [21]). Tertiary preventive interventions aim to treat health problems (e.g. [22]). Giga, Cooper and Faragher [23] found that most common stress management interventions are ‘secondary preventive’, aimed at the individual level and comprised stress management and coping techniques. The same holds true for stress management interventions in the educational sector. Until now stress management interventions in education have been ‘secondary preventive’ mostly and targeted at the individual level [24-28]. These interventions [24-28] all aimed to enhance the individual capacity of (trainee) teachers or teaching assistants to cope with stressors in the workplace, for example via mindfulness-based stress reduction or workshops on stress management skills. However, these interventions were only partly effective in influencing (dimensions of) burnout [24-28] and well-being [28]. More specifically, none of the studies influenced all three burnout dimensions positively, some influenced two dimensions (but always in differing combinations) and the long term effects were not measured. Apparently it is insufficient to reduce burnout and increase well-being in education, by focusing solely on strengthening the individual teachers’ capacity to cope with or manage stress.

### The need for primary preventive organizational interventions and appropriate evaluation studies

The above leads us to the proposition that to decrease (precursors of) burnout, problems should be altered at the source, that is the (interpersonal) work context [8,29], and targeted at the organizational level. This proposition is amplified firstly by the enormous body of research that points to the importance of the (interpersonal) work context in the development of a disbalance between demands and resources (e.g. [30,31]).

Secondly, McVicar, Munn-Giddings, & Seebohm [32] found that primary preventive interventions can take the complexity of an organization into account when designing a preventive strategy. These interventions are therefore potentially more effective than individual level interventions [32].

A review has suggested that if an intervention is effective, the organizational level ones are more likely to bring about positive changes than the individual level ones [33]. On the other hand, two meta-analyses on stress management interventions have failed to show substantial effects of organizational level interventions over individual level interventions [34,35], but this has partly been explained by the underrepresentation of organizational outcome evaluation [35]. For another part, it might be explained by the finding that 'organizational-level occupational interventions are often complex programs involving many people and several intervention components, which might […] complicate the implementation process and the measurement of effects’ ([36], p.85). These interventions thus impose specific demands on the design of the evaluation study (e.g. monitoring the implementation process), demands that cannot be fulfilled by the gold standard design for experiments: the randomized controlled trial [37]. An organization is no laboratory where all conditions can be controlled. However, Griffiths [38] points out that occupational health interventions are still mostly regarded as experiments, set up to discover whether changes occur after manipulating a variable or introducing a particular treatment. Experiments focus on *what* works, thereby discarding to describe the processes which brought about these outcomes (*how* and *why* does it work?) [37]. Nielsen and colleagues [37] posed, that there is a lack of interventions that combine process measures (e.g. managerial support for the intervention) and effect measures (e.g. job demands). To further understand the ‘black box’ and increase the external validity (or generalizability) of interventions, the intervention ought to be evaluated by means of mixed methods [37].

The above underlines the need for appropriate evaluation of primary preventive organizational interventions. This implies for the evaluation study in the current project that: 1) the evaluation design is as rigorous as possible, 2) the implementation process is monitored by assessing process variables, 3) in the analyses it will be assessed how process variables influence intervention outcomes, and 4) (objective) organizational outcomes are measured.

### Effective ingredients of primary preventive organizational interventions

**Figure 1:**
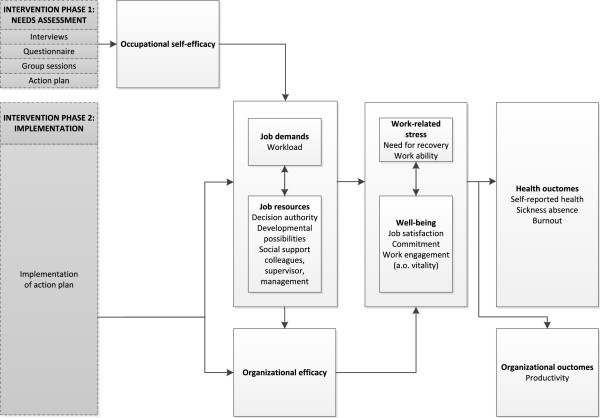
Conceptual model.

The above outlines the need for primary preventive organizational interventions and a mixed methods evaluation, comprising both process and effect measures. But, what components should the intervention, or its application, comprise in order to be effective? In other words, what are effective ingredients for primary preventive organizational interventions in the educational sector? To the best of our knowledge, the current study is the first of that type in that sector. Therefore, we could only argue theoretically what would be the effective ingredients that bring about the desired effect. We propose hereafter that joint ownership (i.e. participation) and occupational self-efficacy play an important role in bringing about the effect on job demands and resources, and need for recovery and vitality (Figure 1).

First, the intervention should be designed in a manner that resembles the tradition of participatory action research (PAR) [39]. Essential in using PAR to design effective stress interventions in the workplace is active participation of stakeholders and (long term) collaboration between researchers and stakeholders [32]. By establishing a participatory group and making use of management’s and worker’s knowledge, skills and perceptions, a feeling of joint ownership of both problems and solutions is created and the participants learn-by-doing how to discuss issues in the workplace. Therefore the intervention should be considered ‘bottom-up’. Nielsen and colleagues have pointed to the relative importance, but rare discussion of joint ownership [40]. This intervention will contribute to that discussion.

Second, the intervention should target occupational and organizational efficacy. Self-efficacy is ‘the belief in one’s own ability to master specific domains in order to produce given attainments’ [41-43]. Occupational self-efficacy refers to beliefs in one’s own ability in the specific domain of work. Self-efficacy can be enhanced in several manners, but the most effective way is through mastery experiences [44]. By taking part in the intervention, it is assumed that workers experience mastery and self-efficacy is thus influenced. A recent study showed that job demands and job resources partially mediated the relation between occupational self-efficacy (or: work self-efficacy) and burnout [45]. The intervention should elaborate empirically on the results of the Consiglio and colleagues article [45]. There is an intervention that comprises these supposedly effective ingredients. The intervention has been developed by a Dutch consulting firm and applied over a hundred times to public and private organizations in The Netherlands in the past decade. That intervention will be tested in the current study.

In sum: we propose an organizational level, primary preventive stress management intervention, aimed to alter the sources of work-related stress by changing the design, management and organization of work [46,47] and to be evaluated by an effect evaluation including organizational outcomes and a process evaluation including process variables related to intervention outcomes. Both the bottom-up intervention, as well as the mixed methods design make this study innovative and a contribution to existing knowledge to the field of organizational interventions.

### Study objectives

The current study tests a participatory, primary preventive organizational level intervention (i.e. a participatory action approach) that targets and engages all workers in the primary process of schools. Participation of employees and managers is supposed to result in increased occupational self-efficacy and organizational efficacy. The application of the intervention will yield work-oriented solutions tailored to the school setting, changing (the balance between) specific job demands and job resources. By improving the balance between job demands and job resources, it is expected to improve precursors of burnout (i.e. high need for recovery, low vitality) in the long run. The central research question is thus: is an organization focused stress management intervention based on participatory action effective in reducing the need for recovery and enhancing vitality in school employees in comparison to business as usual?

In this article we present the design of a controlled trial in two vocational schools in the Netherlands, wherein the participatory action approach and resulting work-oriented solutions are tested empirically.

## Methods/Design

**Figure 2:**
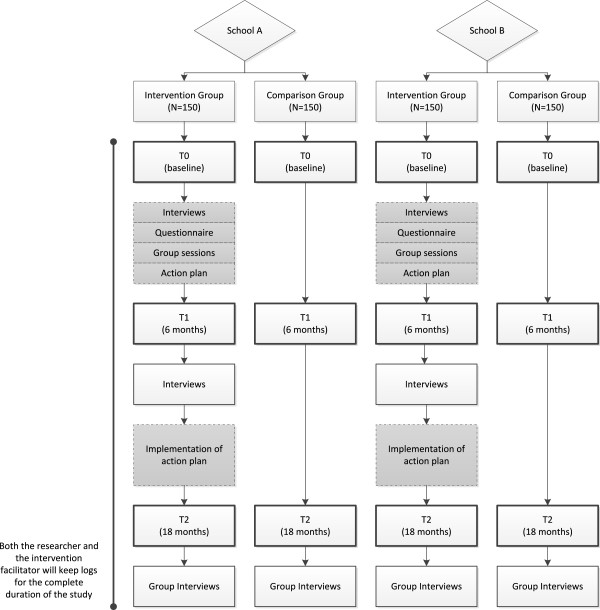
Flow chart of design, measurements, population and intervention program.

A quasi-experimental field study is conducted to determine the effectiveness of the participatory action approach (phase 1: needs assessment) and tailored work-oriented solutions (phase 2: implementation plan), compared to business as usual. The study is designed as a controlled trial (CT) with mixed methods (quantitative and qualitative) and three measurement moments: T0 at baseline (quantitative measurement), T1 at six months (quantitative and qualitative measurement) and T2 at 18 months (quantitative and qualitative measurement) (Figure 2). A CT is necessary to control for random changes, although the researchers are well aware of the fact that they are conducting a *social* experiment and that causal relations are thus embedded in complex contexts [38]. Randomization to experimental group (intervention group) or control group is practically impossible in this project, as often in organizational level workplace interventions [38], due to the aspirations of participating schools. Both schools participate in the study because they aim to solve a problem or reach a goal within a specific department of the school. The experimental groups were thus selected by the schools. To reduce the negative impact of selection bias, the control groups are selected by the researchers according to the ‘general control’ matching principle (or: frequency distribution control) [48]. Matching criteria are: department size (at least 150 employees), mean age of employees, and type of work (i.e. teaching vocational students and not secondary school pupils). Since the assignment to groups was out of our control, we will examine in the analyses whether propensity score matching is necessary. By applying the statistical technique of propensity score matching, the effect of the intervention can be estimated accounted for covariates that predict receiving the intervention. This way we expect to nullify potential confounding bias [49].

### Setting

The project is conducted in two institutions for vocational education (in Dutch: *Middelbaar Beroepsonderwijs* (MBO)) in the western (Alkmaar, Hoorn and Egmond) and northern (Leeuwarden and Heerenveen) Netherlands.

#### Study population

The intervention is applied to one department in both schools, another department in the same school is matched by the researchers as a control group. The target group of the project are teaching and non-teaching (i.e. educational and administrative support staff) employees in two vocational education institutions and their managers. Employees who work within the vocational institution, but do not teach at a secondary vocational level are excluded from the study population (e.g. teachers in general secondary education for adults). All participants are asked to sign an informed consent at baseline.

#### Sample size

The sample size calculation is based on the number of cases required to detect a small (Cohen’s d = 0.2) effect on the primary outcome vitality, as measured with the 3-item subscale of the 9-item version of the Utrecht Work Engagement Scale (UWES-9) [50]. The baseline mean vitality score (range 0–6) is assumed to be 4.01 (SD = 1.14), based on the scores of 9,679 Dutch and Belgian employees [51]. A 5% increase of the mean score on vitality in the intervention group after 12 months is considered relevant and feasible (4.21; SD 1.20).

The required sample size is then 385 (193 for both intervention and control group; thus 97 per intervention and control group per school), assuming a significance level (α) of 0.05, two-sided tests and power (1-β) of 0.80 [52]. A non-response and loss to follow-up of 35% is taken into account, so that a total sample size of 600 is needed.

### The intervention

The intervention that will be tested in this study, named Heuristic Method (HM), is a participatory action approach for diagnosis, development and implementation of workplace interventions [53]. HM has been developed and applied by a Dutch consulting firm in at least 100 public and private organizations in the last decade. The consulting firm refined the intervention after each application, based on the lessons learned. Although the customers were almost always satisfied with the intervention’s results, the intervention effects were never tested scientifically. The Heuristic Method is aimed at optimizing occupational self-efficacy and organizational efficacy. The purpose is to (1) use both management’s and worker’s knowledge, skills and perceptions to thoroughly determine what hinders and stimulates ‘healthy and happy working’ in the organization, so that (2) management and workers can develop their specific work-related action plan and implementation plan that ultimately will reduce need for recovery and increase vitality. The first part of this purpose is addressed in the first phase of the intervention, named ‘needs assessment’. The second part of this purpose is addressed in the second phase of the intervention, the ‘implementation plan’. The needs assessment phase comprises three iterative steps led by an HM-facilitator: (1) interviews; (2) digital questionnaire; (3) group sessions, resulting in a plan of action. The components of the implementation phase can differ according to the maturity of the organization in applying organizational change processes. The minimum variant is remote counseling of the management team by a facilitator, in implementing the plan of action. Details on the application of both phases and their consequent steps in this study are provided below.

#### Phase 1: Needs assessment

The needs assessment is conducted in the tradition of participatory action research (PAR) [39]. Therefore, a participatory group of employees is constituted, comprising employees, a representative from the Workers Council, a staff member, a management member, the HM-facilitator and the researcher (six to eleven members in total). Its members are selected by the management team (with the exception of the facilitator and the researcher), based on their perception of the member’s capacity for ‘pioneering’ in organizational change processes. The HM-facilitator is an expert in organizational change processes. If the intervention group is scattered among several school locations, this will be taken into account when composing the group. The participatory group is named ‘Engine of Development’ and becomes the project’s ambassador throughout the needs assessment. The Engine of Development decides in collaboration how often they meet, but at least six times – before, during and after the three needs assessment steps.

The intervention kicks off with an information session, held after baseline measurement, led by the HM-facilitator and facilitated by the management. In the information session the HM-facilitator outlines the steps of the upcoming intervention and the researcher presents several outcomes of the baseline measurement.

##### Step 1: In-depth interviews

The Engine of Development approaches some prominent colleagues for an in-depth, open interview with the HM-facilitator. Prominent colleagues can be the typical optimists, pessimists, innovators, integrators or otherwise interesting employees that help the HM-facilitator grasp both initial hindrances to happy and healthy working as well as implicit norms in the intervention group. Approximately ten interviews will be held, or until saturation is reached. The HM-facilitator writes a report on his findings that is sent to all employees, after consulting the Engine of Development and the management team, respectively.

##### Step 2: Digital, open ended questionnaire

Based on the report of the in-depth interviews, the Engine of Development compose the questions for a digital, open ended questionnaire. The open ended questionnaire should further specify the hindrances that were found in the in-depth interviews. All employees in the intervention group are invited to take part in the questionnaire. The HM-facilitator writes a report on the findings that is sent to all employees, after consulting the Engine of Development and the management team, respectively.

##### Step 3: Group sessions

Based on the report of the open ended questionnaire, the Engine of Development determines several clusters of hindrances that need to be specified further in group sessions. The aim of the group sessions is not only to specify hindrances, but also to propose work-oriented solutions. All employees in the intervention group are invited to take part in the group sessions, which are chaired by the Engine of Development (except for the researcher). To ensure ‘freedom of speech’, the management team and staff have their own group sessions.

##### Result: Plan of Action

The HM-facilitator adds his own observations, experiences and assessment of (un)healthy implicit norms in the intervention group to the compressed analyses of hindrances and solutions offered in the in-depth interviews, open questionnaire and group sessions. This results in a plan of action that supposedly reflects opinions, perspectives and (feasible) wishes of employees in the intervention group (i.e. management and workers). The plan of action is sent to all employees, after consulting the Engine of Development and the management team, respectively. If the work-oriented solutions proposed also concern higher management (i.e. Executive Board or Board of Directors), then the management team is advised to inform higher management on the findings as well.

#### Phase 2: Implementation

In the implementation phase of the participatory action approach, the management has to take the first step. With support of the Engine of Development, the management team decides on which work-oriented solutions will be implemented. The solutions can be either evidence-based (e.g. adjusting classroom schedules), or new, tailor-made and specific to the context (e.g. adjusting the physical layout of class rooms). In any case, the management team explains to the employees which work-oriented solutions are (not) implemented and to what end. Furthermore, the method prescribes that the management team equips the plan of action with an implementation plan comprising amongst others a timeframe, a budget and the allocation of roles (e.g. the Engine of Development’s role). If the management team wishes, the HM-facilitator will take up a role during the implementation phase, for example in monitoring the plan’s progress or coaching the management team.

### Primary and secondary outcomes

#### Measuring instruments: primary outcomes

##### Need for recovery

(Early) symptoms of fatigue at work are considered indicative of a ‘need for recovery’ [54]. Need for recovery after a working day is measured using a subscale of the Dutch Perception and Evaluation of Work Questionnaire (Dutch abbreviation: VBBA) [54]. The questionnaire comprises 11 dichotomous (*yes/no*) items and has proven to be valid and reliable (alpha 0.86) [54].

##### **Vitality**

Vital workers show high levels of energy and mental resilience, persist when facing difficulties and are willing to invest effort in their work [50]. Vitality is measured using the vigor subscale (3 items) of the Utrecht Work Engagement Scale-9 [55]. Responses are to be given on a seven point scale (0 = *never* to 6 = *always/every day*). The vigor subscale has shown acceptable validity and reliability in a sample across ten countries [50].

#### Measuring instruments: secondary outcomes

##### Job demands and resources

Several aspects of the job and its content are measured using subscales of the Dutch version of the Job Content Questionnaire (JCQ) [56]: psychological demands, coworker and supervisor support and decision authority. The psychological demand dimension measures the mental work load, organizational constraints on task completion and conflicting demands (5 items) [56]. The coworker and supervisor support subscales measure socio-emotional as well as instrumental support (8 items) [56]. Four identically phrased, but explorative items were added on socio-emotional and instrumental support of (higher) management. Decision authority or autonomy measures the workers’ possibilities to make decisions about their work, mediated by organization factors (3 items) [56]. Response scales range from 1 = *strongly disagree* to 4 = *strongly agree.* The subscales of Dutch JCQ used in this study have shown acceptable scale reliability and validity [56].

Furthermore, possibilities for professional growth are assessed by a 6-item subscale of a Dutch questionnaire, developed for the (primary) educational sector (in Dutch: Welzijnscheck Onderwijspersoneel) [57], which has shown good divergent validity and reliability (alpha 0.87) [58]. Response scales range from 1 = *strongly disagree* to 5 = *strongly agree.*

##### Work ability

Work ability is based on the workers’ balance between resources and demands and determines job performance now and in the near future [59]. Work ability is measured using the Work Ability Index (WAI) [60]. The WAI is a self-report instrument and comprises seven dimensions on the physical and mental demands of work and the health and resources of the employee. For the current study, two of the seven dimensions were deemed relevant: 1) perceived current work ability, compared to lifetime best (1 item) and 2) perceived work ability related to mental job demands and perceived work ability related to physical job demands (2 items). Responses on (1) are recorded on a frequency scale from 0 (*unable to work*) to 10 (*very good)*. Responses on (2) are recorded on a five-point frequency scale from 1 (*very good*) to 5 (*very bad*). Reliability and validity have been shown to be adequate in a Dutch sample (alpha 0.63 to 0.71) [61].

##### Job satisfaction

Job satisfaction is operationalized as workers’ satisfaction with the job and its conditions. Job satisfaction is measured by two items of the Netherlands Working Conditions Survey 2010 [62]: to what extent are you, all things considered, satisfied with (1) your job, and (2) your working conditions? Response scales range from 1 = *very dissatisfied* to 5 = *very satisfied*.

##### Commitment

It has been shown that teacher commitment is a predictor of burnout, sickness absence and retention [63]. Therefore, commitment to work (2 items) and the organization (3 items) is measured in this study, using the Dutch questionnaire NOVA-WEBA [64,65], which has shown moderate validity and reliability (alpha 0.68) [66]. Response scales range from 1 = *strongly disagree* to 5 = *strongly agree*.

##### Work engagement

Work engagement is defined as a positive, fulfilling, work-related state of mind that is characterized by vigor, dedication and absorption [55]. The Utrecht Work Engagement Scale (UWES) [55] is the most commonly used instrument to measure work engagement [67]. The 9-item version of UWES is used in this study, response scales range from 0 = *never* to 6 = *always/every day*. UWES-9 has shown good validity and reliability [68].

##### Health

Health is measured by asking a single item of the free version of SF-36-v2, named RAND-36 [69]. This single item measures perceived general health (“How do you rate your health in general?”) on a five-point frequency-scale from 1 = *bad* to 5 = *excellent*. The subscale is considered valid and reliable (alpha 0.81) [69].

##### Sickness absence

Sickness absence is considered working less than normal hours or days due to illness, an incident or any other health reason. Sickness absence data will be collected in two ways: from company records as well as at baseline and follow-up measurements.

At baseline and follow-up, presence, frequency and duration of sickness absence in the past 12 months is measured by three items from the Netherlands Working Conditions Survey 2010 [62]. Furthermore, the self-reported cause of the last case of sickness absence is measured and whether this cause was attributed (fully, partly or not) to the work (NWCS 2011) [3].

##### Burnout

Burnout is measured with a slightly adjusted, Dutch version of the Maslach Burnout Inventory-General Survey (MBI-GS) [70], named Utrecht Burnout Scale (UBOS) [71]. This 16-item questionnaire includes the key dimensions of burnout: emotional exhaustion (feeling drained by the work), depersonalization (a cynical attitude towards the work and people working with) and reduced personal accomplishment (feeling incompetent at work). Response scales range from 0 = *never* to 6 = *every day*. Several studies have shown that the MBI-GS and its subscales are excellently reliable and valid (e.g. [72,73]).

##### Inrole performance and knowledge and skills

Inrole performance is considered the achievement of work-related goals and measured by three items of the Netherlands Working Conditions Survey 2010 [62], with a response scale ranging from 1 = *strongly disagree* to 5 = *strongly agree*. Furthermore, the fit between current knowledge, skills and job tasks is measured by asking one item from NWCS 2011 [3]: “How do your knowledge and skills fit your current work?”. Response scales range from 1 = *less knowledge and skills than needed*, to 2 = *it fits* to 3 = *more knowledge and skills than needed*.

##### Willingness and ability to prolong working life

The willingness and ability to prolong working life is measured by asking two open ended items from the Netherlands Working Conditions Survey 2010 [62]: Until what age do you (1) think you are able to continue working, and (2) want to continue working?

##### Productivity

Individual productivity in work is measured by a single item, based on module E of the PRODIDSQ [74]. PRODISQ is a scale considered to facilitate the validity of productivity costs estimates [75]. This single item measures self-rated productivity (“How would you assess your overall work performance in the past 4 weeks on a scale of 0 to 10?”) from 0 = *worst quality* to 10 = *best quality*.

### Measuring instruments: mediating factors

#### Occupational self-efficacy

Occupational self-efficacy is described as the confidence a worker has in his or her perceived ability to perform job tasks successfully [76]. The short (6 item) version of the Occupational Self-efficacy scale [77] measures the concept in a valid and reliable way (alpha 0.85) on a five-point Likert scale from 1 = *totally disagree* to 5 = *totally agree*[76].

#### Organizational efficacy

Organizational efficacy is defined as ‘an individual’s perception of the general capabilities of an organization’ ([78], p. 127). Van Vuuren’s seven item Organizational Efficacy Scale (OES) [78] has shown to measure the concept reliably (alpha 0.81) on a five-point Likert scale (1 = *strongly disagree*, 5 = *strongly agree*).

### Measuring instruments: sociodemographic and profiling data

Sociodemographic data are collected at baseline, i.e.: gender; age; level of education; household composition; working hours per week; number of years working in current function, school and sector; main workplace location.

Profiling data are collected either at baseline or at follow-up measurements for a practical reason. A practical reason is that response rates would drop if questionnaires would be too intrusive. Profiling questions are considered stable over time, which makes the measurement moment less important.

#### Locus of Control

Locus of control is considered a personality trait and defined as the extent to which people believe they can influence the course of their lives [79]. The construct comprises two dimensions - internal and external locus of control - and can be measured with 8 items on a seven-point Likert-scale (1 = *totally disagree* to 7 = *totally agree*) [80]. The subscale internal locus of control has shown poor reliability (alpha 0.43) and the subscale external locus of control has shown moderate reliability (alpha 0.66) in a German population of youngsters [80]. The replication of the theoretical two factor structure in an exploratory factor analysis, indicates that the instrument’s validity might be more promising [80]. Despite the problematic reliability, the Nolte-scale was preferred over the original I-E scale by Rotter (23 items) [79], because it is more compact. The Nolte-scale is translated to Dutch by a native German speaker living in The Netherlands for long and then back-translated to German by a native Dutch speaker living in Germany for long.

#### Parent and pupil (mis)behavior

The extent to which employees are bothered by (mis)behavior of pupils and parents in their work, is measured by a Dutch scale designed for the (primary) educational sector, named Welzijnscheck Onderwijspersoneel [57]. Misbehavior of pupils is measured reliably (alpha 0.82) with six items on a six-point scale ranging from 1 = *not applicable* to 6 = *in a very great degree*) [58]. On the same six-point scale, misbehavior of parents is measured reliably (alpha 0.78) with four items [58].

#### Work-life interference

Work-life interference is measured by one explorative item (“Does your work interfere with your private life?”) as well as life-work interference (“Does your private life interfere with your work?”), on a five point Likert scale ranging from 1 = *almost never* to 5 = *almost always*.

#### Institutional policy and educational quality

Workers’ knowledge of institutional policies is measured by one explorative item (“I am aware of the policies of my organization”) on a five-point Likert scale ranging from 1 = *strongly disagree* to 5 = *strongly agree*. The workers’ perception of the educational quality is also measured by one explorative item (“Our school prepares participants well for professional practice”) on a five-point Likert scale ranging from 1 = *strongly disagree* to 5 = *strongly agree*.

### Data analysis

#### Effect evaluation

The effectiveness of the intervention on primary outcomes (need for recovery and vitality) and secondary outcomes at short term (T1), long term (T2) and corrected for baseline values, will be established by multilevel analyses. Repeated measurements on the worker-level and clustering of observations can thus be taken into account. The data will be analyzed at three levels: 1) worker, 2) department, and 3) school. Both crude and adjusted linear and logistic regression analyses will be conducted. The intention-to-treat principle is leading in all statistical analyses, meaning that the analyses are based on the initial treatment assignment and not on the treatment eventually received. However, per-protocol analysis will also be conducted, restricting the comparison to the ideal participants, in this study: participants that report taking part in at least two of the three steps of the needs assessment.

Multilevel analyses wherein T1 functions as the dependent variable, will be adjusted for possible confounding factors (e.g. experience, overtime). These variables will also be checked for effect modification at all measurement moments.

For all analyses, a two-tailed significance level of p < 0.05 will be considered statistically significant. The multilevel analyses will be conducted by means of MlwiN 2.0; linear and logistic regression analyses will be performed using SPSS 17.0 (SPSS Inc. Chicago, Illinois, USA).

### Process evaluation

An extensive process evaluation will be conducted based on two complementary pillars: 1) Stecklar and Linnan’s framework [81] and its adaptations by Murta, Sanderson and Oldenburg [82], and 2) a selection of Randall, Nielsen and Tvedt’s Intervention Process Measure (IPM) [83]. The first pillar helps to answer instrumental questions concerning the intervention process (how does the intervention work?). The second pillar helps to identify participants’ appraisals of the intervention process (why does the intervention work?). A combined approach seems necessary to answer both the “how” and “why” question, since some studies have shown that an equal amount of ‘dose received’ can yield a range of heterogeneous individual appraisals of the intervention [84]. And the appraisal of intervention processes may in turn influence intervention outcomes [85].

Stecklar and Linnan [81] propose seven components to determine how the intervention was implemented (Table 1): (i) recruitment (what procedures were used to interest workers and what are reasons for not participating?), (ii) reach (attendance of workers in each phase of the participatory approach and its consequent tailored measures), (iii) dose delivered (how many steps of the participatory approach were actually delivered by the facilitator?), (iv) dose received (how many steps of the participatory approach were actually followed by the worker?), (v) fidelity (was the participatory approach delivered according to protocol?), (vi) satisfaction (how satisfied are participants with the participatory approach?); and (vii) context (what organizational and environmental characteristics affect the intervention?).

**Table 1:** Process evaluation components and (examples of) questions

**Ref.**	**Components**	**Questions**	**Logs researcher**	**Logs facilitator**	**Interviews**	**T1**	**Group interviews**	**T2**
[81,82]	*Recruitment*	what procedures were used to interest workers and what are reasons for not participating?	X					
[81,82]	*Reach*	attendance of workers in each phase of the participatory approach and its consequent tailored measures			X	X	X	X
[81,82]	*Dose delivered*	how many steps of the participatory approach were actually delivered by the facilitator?	X		X			
[81,82]	*Dose received*	how many steps of the participatory approach were actually followed by the worker?		X		X		
[81,82]	*Fidelity*	was the participatory approach delivered according to protocol?	X		X	X	X	X
[86]	*Satisfaction*	how satisfied are participants with the participatory approach?			X	X	X	X
[84,86]	*Context*	what organizational and environmental characteristics affect the intervention?	X		X		X	
[84]	*Line manager attitudes and actions*	e.g. “My immediate manager has done a lot to involve employees throughout the process”				X		X
[84]	*Exposure to components of the intended intervention*	e.g. “The project has made it easier to tackle the changes in the organization”				X		X
[84]	*Employee involvement*	e.g. “I had the opportunity to give my views about the change before it was implemented”				X		X
[84]	*Employee readiness for change*	e.g. “I looked forward to the changes brought about by the project”				X		X

Randall and colleagues [83] have shown that participants’ appraisals of an intervention and its implementation can be measured quantitatively by the five scales of the Intervention Process Measure, of which four are used and adjusted to fit this study (Table 1): (a) line manager attitudes and actions, (b) exposure to components of the intended intervention, (c) employee involvement, and (d) employee readiness for change. Scores on these scales will be related to intervention outcomes (e.g. job satisfaction, well-being, and self-efficacy).

Six sources of data are used to assess the proposed process aspects (Table 1): (1) data logs by the researchers (recruitment, dose delivered, fidelity, context); (2) logs by the facilitator (dose received); (3) interviews at T1 with employees, management, participatory group and facilitator (reach, dose delivered, fidelity, satisfaction, context); (4) questionnaire at T1 (reach, dose received, fidelity, satisfaction, line manager attitudes and actions, exposure to components of the intended intervention, employee involvement and employee readiness for change; (5) separate group interviews with employees and management at T2 (reach, fidelity, satisfaction, context); (6) questionnaire at T2 (reach, fidelity, satisfaction, line manager attitudes and actions, exposure to components of the intended intervention, employee involvement and employee readiness for change). Data will be analyzed by either qualitative data software (e.g. Kwalitan) or by using a qualitative rating procedure.

### Ethical considerations

The study protocol and materials are approved by TNO’s Review Committee Participants in Experiments (RCPE), an internal ethics committee that assesses ethical aspects of working with participants in experiments. After review, the committee stated that in this study “the information is complete, participants can join voluntarily and an informed consent is provided”. The RCPE has thus given a positive advice to the study’s responsible manager, who decided to follow the positive approval by giving permission for performing the study. Hereafter we will elaborate on the information provision prior to and during the study.

#### Information provision prior to the study

Prior to the study, higher management of both schools signed a letter of intent to participate, cooperate and invest in kind. At the start of the project, a more detailed project plan will be presented to higher management in both schools where after working arrangements are made.

#### Information provision on measurement

Prior to baseline measurement employees in the intervention and control group will receive verbal and written information on the baseline measurement. Employees in the intervention group will be informed verbally by the participatory group (Engine of Development) by presentations at team meetings. Employees in the control group will be informed by their immediate supervisors during team meetings. Management in both the inter2vention and control group will be informed by the facilitator during a management meeting. All groups will receive a digital letter with information about the baseline measurement.

Furthermore, the agenda of a team meeting will be cleared, to enable employees to fill out the questionnaire(s) during this time frame.

All employees and managers participating in the baseline measurement are requested to sign an informed consent at the start of the questionnaire. By signing the informed consent participants declare amongst other things: 1) to have received information on the baseline measurement and the study, 2) to understand they can withdraw at any time from the study without reason. A similar procedure will be applied for the first follow-up (T1, 6 months) and second follow-up (T2, 18 months).

#### Information provision on participatory approach

Then, for both intervention groups a briefing will be held to announce the start of the participatory approach. During the briefing, the participatory approach is explained by the facilitator and the role of all participants is clarified. All questions can be asked. In addition, some of the results of the baseline measurement will be fed back during the meeting. The researchers’ prior experiences in educational institutions have learned that an enormous general skepticism towards survey research has to be overcome before support can be created. Demonstrating that results of the questionnaires are actually used for the better, helps to create support.

#### Information provision during the participatory approach

Continuously informing employees on the progress of each step is inherent to the participatory approach. The proceedings of each step are fed back to all staff of the intervention group, after suggestions from the participatory group and management are taken into account.

## Discussion

In the educational sector, job demands have intensified at rapid pace in recent years, while job resources remained the same. The imbalance between demands and resources contributes to the development of mental health problems such as burnout. At the organizational level burnout resonates in increased sickness absence rates and problems with retention of experienced and novice teachers. Until now, most intervention studies that aimed to target these problems have been only partly effective, possibly because they focused on the individual level and applied secondary preventive interventions. Instead, it has been argued theoretically to focus on the organizational level and application of primary preventive interventions. The current research helps to translate this theoretical reasoning to empirical studies.

This is the first study to describe the test of a participatory, primary preventive organizational level intervention (i.e. a participatory action approach) on work-related stress and well-being that targets and engages all workers in the primary process of vocational education training schools. The goal of this study is to determine whether the participatory action approach, which is supposed to result in tailored, work-oriented solutions on the balance between job demands and job resources, effectively influences need for recovery and vitality.

### Strengths and limitations of the intervention

Less than a quarter of the intervention studies focus on primary preventive interventions, as assessed in a recent meta-analysis on stress interventions [35]. Thus, a first strength of the current intervention is the aim to alter job stress at its core (primary prevention).

Second, by making use of the participatory action approach, stakeholders at all levels are involved - teachers in the first place. This ‘bottom-up’ involvement of all stakeholders likely contributes to commitment to the proposed solutions. Solutions that can count on both the management and the work floor’s commitment are more sustainable and thus more likely to have impact [37,40].

A third strength is that the intervention is conducted in the same way for both schools, but the tailored workplace solutions can differ. This way we can compare different solutions on similar outcomes, further contributing to evidence-based practice.

Fourth, the project requires close collaboration between the intervention provider (i.e. the facilitator) and the researcher. They have a different task to fulfill within the project: the facilitator needs to make the intervention work, the researcher needs to make the study design work. Inevitably, their world views (or: paradigms) will meet and, maybe clash. An issue is that the intervention provider works from a ‘practice-based evidence’ perspective, asking himself at every step: is it useful? Is it important? Is it valid? Whereas researchers aim to contribute to evidence-based practice and therefore ask themselves: is it valid? Is it important? Is it useful? [84]. Even though the differing paradigms and activities will probably make the project difficult from time to time, the primary interest of both intervention provider and researcher is eventually the same: working happier and healthier in vocational education.

#### Strengths and limitations of the study

In line with earlier recommendations (e.g. [86]) the current study assesses all steps of the intervention process, that is: 1) the intended intervention, 2) intended changes in exposure or behavior (i.e. job demands and job resources), and 3) intended changes in study outcomes (i.e. need for recovery and vitality). From an (occupational) epidemiological point of view, the study can be classified as a *prevention-effectiveness study*[86,87] as opposed to the *etiologic intervention study* (i.e. the most rigorous epidemiologic design, derived from the controlled clinical trial, studying disease and health outcomes [87]). Characteristics of the prevention-effectiveness study design are amongst others: small samples, no randomization or blinding, test of a program theory, quantitative and qualitative measures, case studies [88]. These characteristics ensure the internal validity of the study. Prevention effectiveness trials are at the core of evidence-based public health [87,88]. The results of our study will be fed back to the vocational education council and policy makers in the field, helping them to ‘practice evidence-based’. From a sociological, psychological or anthropological point of view, our study would rather be classified as a *pragmatic trial*, designed to find out ‘how effective a treatment actually is in routine, everyday practice’ [89]. Since we are conducting research in practice, several unforeseen events (e.g. reorganization) can take place, which might endanger the feasibility of the study. But our study is designed to reflect what happens in ‘the real world’ [89], maximizing external validity. This asks of the researchers and intervention providers to adjust to unexpected changes, while respecting the ‘intention-to-treat-principle’ (i.e. once allocated to the intervention or control group, always allocated to intervention or control group). Pragmatic trials allow for subtle variations in the intervention and research protocol, so to match the schools’ context and needs. The researchers will not permit any variations in the protocol of the first phase of the intervention (needs assessment), but broad variations are permitted in the second phase of the intervention. In the current study, points of view from the epidemiological, sociological and psychological disciplines are combined, so to maximize both internal and external validity [90].

A strength of the study is that both psychological outcomes (e.g. job satisfaction) as well as organizational outcomes (e.g. sickness absence) are taken into account, contrary to most intervention studies published [35]. The outcomes are assessed qualitatively (i.e. (group) interviews, observations, logs) and quantitatively (i.e. self-report measures in digital survey) and whenever possible complemented with ‘objective’ organizational data (e.g. sickness absence registration).

Besides strengths, the study also comprises possible limitations. A limitation of this study is the quasi-experimental design. However, the study’s design – a controlled trial with departments allocated to conditions and two follow-up measurements - is as rigorous as possible in a practice-based study in (vocational) education. On the one hand, by giving higher management a vote in the choice for the intervention group, selection bias is possibly introduced. On the other hand, higher management’s commitment to the study is assured and thereby relevance and feasibility of the study. A second limitation is the timeframe of the study. Behavioral and organizational changes do not come easy nor quickly. Therefore, the timing of follow-up measurements (six and eighteen months) might be too soon to establish the organizational changes and detect effects.

To conclude, Kristensen [86] reminds occupational intervention researchers of the ‘simple fact that the purpose of workplaces is to produce goods and services – not to serve as arenas for intervention research’. The “Bottom-up Innovation” project will probably encounter numerous unexpected changes, but the design and research methods are chosen carefully, so to optimize both internal and external validity.

## Abbreviations

CT: Controlled trial; HM: Heuristic method (i.e. the intervention); IPM: Intervention process measure; JCQ: Job content questionnaire; MBI-GS: Maslach burnout inventory-general survey; NOVA-WEBA: Dutch questionnaire developed to identify risk factors for work stress; NWCS: Netherlands working conditions survey; OES: Organizational efficacy scale; PAR: Participatory action research; RAND-36: General health scale; PRODISQ: PROductivity and DISease Questionnaire; RCPE: Review committee participants in experiments; SMI: Stress management intervention; T0: Baseline measurement; T1: First follow-up measurement; T2: Second follow-up measurement; UBOS: Utrecht burnout scale; UWES-9: Utrecht work engagement scale-9; VBBA: The Dutch perception and evaluation of work questionnaire; WAI: Work ability index.

## Competing interests

The authors declare that they have no competing interests.

## Authors’ contributions

RS will conduct the study and was responsible for drafting the paper. JvG wrote the original study protocol, was involved in preparations of the study and provided intellectual input for the article. KOH, NW, BB, EB and AvdB provided intellectual input and had a role in supervision. All authors commented on the draft versions. All authors have read and approved the final version of the manuscript.

## Pre-publication history

The pre-publication history for this paper can be accessed here:

http://www.biomedcentral.com/1471-2458/13/760/prepub
